# Supplementary material for: The Healthfulness of the US Packaged Food and Beverage Supply: A Cross-Sectional Study
Source: Nutrients. 2019 Jul 24;11(8):1704. doi: 10.3390/nu11081704 (PMC6722673; doi:10.3390/nu11081704)

Figure S1. Example of an HSR calculation for a snack bar

Determine the HSR category of the food
- *Category 1 = non-dairy beverages*
- *Category 1D = dairy beverages*
- *Category 2 = foods*
- *Category 2D = dairy foods*
- *Category 3 = fat, oils*
- *Category 3D = high fat cheeses*

Determine the HSR category of the food
- *Category 2 = foods*

Calculate baseline points
- *Energy points based on Energy (kJ) per 100g*
- *Saturated fat points based on Saturated fat (g) per 100g*
- *Total sugars points based on Total sugars (g) per 100g*
- *Sodium points based on Sodium (mg) per 100g*

Baseline points = Sum

Calculate baseline points
- *Energy points: 1700 kJ per 100g = 5 points*
- *Saturated fat: 1.0g per 100g = 0 points*
- *Total sugars: 15.3g per 100g = 3 points*
- *Sodium: 50 mg per 100g = 0 points*

Baseline points = 5 + 0 + 3 + 0 = 8

Calculate modifying points
- *Protein points based on Protein (g) per 100g*
- *Fiber points based on Fiber (g) per 100g*
- *V points based on food type*

Modifying points = Sum

Calculate modifying points
- *Protein: 2.5g per 100g =1 point*
- *Fiber: 6.2g per 100g = 6 points*
- *V points for snack bars = 0 points*

Modifying points = 1 + 6 + 0 = 7

Calculate final HSR Score
Final score = Baseline points – Modifying points

Calculate final HSR Score
Final score = 8 – 7 = 1

Calculate final HSR
HSR based on HSR Score and Category

Calculate final HSR
HSR: HSR Score 1 = 3.5

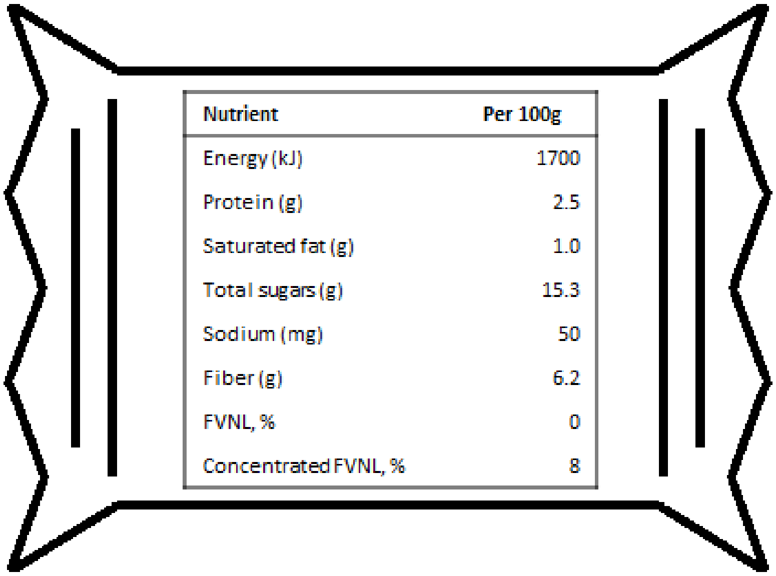

Nutrient	Per 100g
Energy (kJ)	1700
Protein (g)	2.5
Saturated fat (g)	1.0
Total sugars (g)	15.3
Sodium (mg)	50
Fiber (g)	6.2
FVNL, %	0
Concentrated FVNL, %	8

Figure S2. Included and excluded products

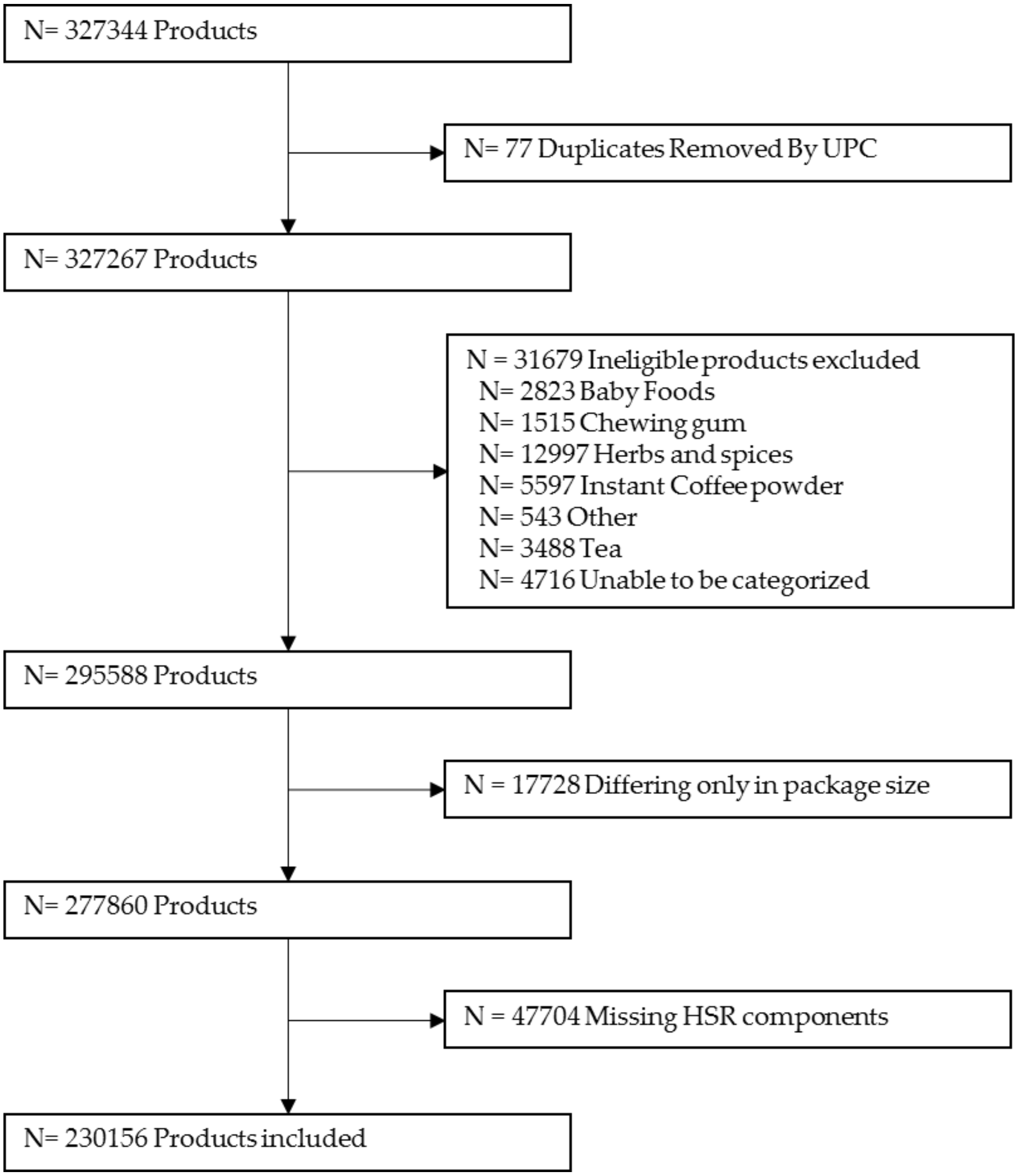

Table S1. Healthfulness of the US food and beverage supply by minor food category.

Minor food category	No. products	Nutrient profiling summary score		Level of processing
		Mean HSR (SD)	Proportion 'healthy' HSR ≥ 3.5 (%)	Proportion ultra-processed (%)
**Eggs**	**487**	**3.9 ± 0.5**	**94.3**	**0.0**
**Fruit, vegetables, nuts and legumes**	**38032**	**3.7 ± 1.1**	**70.3**	**18.3**
Fruit	10407	2.8 ± 1.0	49.0	34.8
Jam and marmalades	2474	2.2 ± 0.5	4.8	100.0
Nuts and seeds	6494	4.1 ± 0.8	85.7	0.0
Vegetables	18657	4.3 ± 0.9	85.5	4.7
**Seafood and seafood products**	**4325**	**3.7 ± 0.8**	**81.7**	**20.3**
**Cereal and grain products**	**18024**	**3.2 ± 1.1**	**52.8**	**61.8**
Breakfast cereals	5334	3.1 ± 1.1	41.1	91.8
Cereal and nut bars	2378	2.4 ± 0.9	15.3	100.0
Couscous	103	3.7 ± 0.9	68.9	0.0
Noodles	576	3.2 ± 1.3	65.3	100.0
Pasta	5196	3.6 ± 1.0	75.2	38.8
Rice	1918	3.2 ± 1.0	60.9	50.6
Other cereals	2519	3.3 ± 1.3	57.1	12.0
**Convenience foods**	**17980**	**3.2 ± 0.8**	**51.9**	**90.9**
Meal kits	102	2.8 ± 0.9	44.1	100.0
Pizza	2364	2.7 ± 0.6	16.5	100.0
Pre-prepared salads and sandwich	2270	3.1 ± 0.7	46.3	100.0
Ready meals	7599	2.6 ± 0.9	27.0	100.0
Ready-made breakfast meals	774	3.2 ± 0.7	60.7	100.0
Soup	4763	3.4 ± 1.1	62.2	65.8
Other frozen foods	108	3.3 ± 0.4	60.2	100.0
**Edible oils**	**3246**	**3.0 ± 1.3**	**55.9**	**8.4**
Cooking oils	2412	3.5 ± 1.0	71.8	0.0
Edible oils	834	1.5 ± 1.1	10.2	32.7
**Foods for specific dietary use**	**2369**	**2.9 ± 1.1**	**41.4**	**100.0**
Breakfast beverages	30	3.1 ± 1.9	63.3	100.0
Diet drink mixes	39	3.7 ± 0.5	97.4	100.0
Protein and diet bars	1630	2.6 ± 1.1	21.3	100.0
Sports gels	43	2.3 ± 0.6	9.3	100.0
Other fitness or diet products	627	3.7 ± 0.7	91.4	100.0
**Dairy**	**27839**	**2.9 ± 1.4**	**40.7**	**61.5**
Cheese	9335	2.9 ± 1.6	48.5	18.1
Cream	904	2.1 ± 1.2	19.0	61.6
Desserts	819	2.2 ± 1.0	7.9	100.0
Ice cream and edible ices	8234	2.3 ± 0.7	8.4	100.0
Milk	4427	3.2 ± 1.5	64.4	67.1
Yogurt and yogurt drinks	4120	4.0 ± 1.0	73.3	69.4
**Snack foods**	**12231**	**2.6 ± 1.2**	**29.6**	**100.0**
**Non-alcoholic beverages**	**19954**	**2.5 ± 1.7**	**35.7**	**83.1**
Beverage mixes	2779	0.8 ± 0.7	1.7	100.0

Coffee and tea	132	2.5 ± 1.8	34.1	100.0
Cordials	68	0.8 ± 0.4	0.0	100.0
Energy drinks	394	1.5 ± 0.5	0.0	100.0
Fruit and vegetable juices	6324	4.5 ± 0.8	95.4	68.0
Soft drinks / sodas	5625	1.3 ± 0.5	0.0	100.0
Sports drinks	302	1.7 ± 0.3	0.0	100.0
Waters	3702	2.7 ± 1.4	26.7	63.4
Other beverages	628	1.5 ± 0.5	0.5	100.0
**Sauces, dressings, spreads and dips**	**21772**	**2.5 ± 1.2**	**32.9**	**92.3**
Mayonnaise and salad dressings	4285	1.9 ± 0.9	14.1	79.6
Sauces	11307	2.3 ± 1.1	23.5	98.7
Spreads	6180	3.2 ± 1.0	63.0	89.5
**Meat and meat alternatives**	**12249**	**2.2 ± 1.3**	**35.7**	**75.3**
Meat alternatives	653	3.9 ± 0.6	91.4	74.9
Processed meat	11596	2.1 ± 1.3	32.6	75.3
**Bread and bakery products**	**30194**	**2.1 ± 1.1**	**20.0**	**98.6**
Bread	8695	3.3 ± 0.8	56.3	95.1
Cakes, muffins and pastries	10318	1.6 ± 0.8	3.5	100.0
Crackers and cookies	11181	1.6 ± 1.0	7.0	100.0
**Sugars, honey and related products**	**4625**	**1.5 ± 0.9**	**6.7**	**73.0**
Dessert additions	1566	1.4 ± 0.8	1.6	100.0
Dessert toppings	319	1.4 ± 0.9	3.8	100.0
Honey	671	1.5 ± 0.2	0.3	0.0
Sugar	577	0.6 ± 0.4	0.5	0.0
Sweeteners	351	2.4 ± 1.2	50.4	100.0
Syrup	1141	1.8 ± 0.7	7.8	100.0
**Confectionery**	**16829**	**1.1 ± 0.7**	**1.4**	**100.0**
Chocolate and sweets	16171	1.1 ± 0.6	0.5	100.0
Jelly	658	1.8 ± 1.4	23.3	100.0

Table S2. Deciles of energy, saturated fat, sodium, and total sugars by minor category in the US food and beverage supply.

Minor food category	Nutrient composition colored by decile (Median (IQR))				Nutrient Decile[1]
	Energy (kcal100g)	Saturated fat (g/100g)	Sodium (mg/100g)	Total Sugars (g/100g)	
**Eggs**	**140 (125-140)**	**3.0 (2.0-3.3)**	**140 (140-163)**	**0.0 (0.0-0.0)**	0
**Fruit, vegetables, nuts and legumes**	**107 (48-421)**	**0.0 (0.0-3.3)**	**63 (4-300)**	**5.6 (2.4-25.0)**	1
Fruit	321 (71-452)	0.0 (0.0-5.4)	13 (0-75)	32.0 (14.1-50.0)	2
Jam and marmalades	250 (200-250)	0.0 (0.0-0.0)	0 (0-28)	50.0 (42.1-60.0)	3
Nuts and seeds	571 (561-607)	6.7 (3.6-7.1)	250 (0-400)	3.6 (3.3-7.1)	4
Vegetables	50 (28-92)	0.0 (0.0-0.0)	182 (18-353)	2.4 (0.8-4.8)	5
**Seafood and seafood products**	**120 (89-189)**	**0.5 (0.0-1.8)**	**375 (223-529)**	**0.0 (0.0-0.9)**	6
**Cereal and grain products**	**368 (353-394)**	**0.0 (0.0-2.3)**	**221 (0-500)**	**4.4 (1.8-23.1)**	7
Breakfast cereals	385 (370-404)	0.0 (0.0-1.8)	346 (107-500)	23.3 (14.3-32.1)	8
Cereal and nut bars	422 (393-457)	4.2 (2.2-7.1)	243 (143-333)	27.5 (21.4-33.3)	9
Couscous	357 (345-368)	0.0 (0.0-0.0)	10 (0-821)	1.8 (0.0-2.3)	
Noodles	375 (346-393)	0.9 (0.0-1.8)	49 (27-784)	3.6 (1.6-3.6)	
Pasta	357 (321-364)	0.0 (0.0-1.5)	9 (0-458)	3.6 (1.8-3.6)	
Rice	356 (333-360)	0.0 (0.0-0.0)	157 (0-911)	0.1 (0.0-2.6)	
Other cereals	367 (344-393)	0.0 (0.0-0.0)	100 (0-833)	3.1 (0.0-6.7)	
**Convenience foods**	**176 (103-247)**	**1.7 (0.4-3.5)**	**384 (265-557)**	**2.1 (1.1-3.6)**	
Meal kits	232 (53-275)	1.7 (0.0-2.8)	592 (45-1143)	2.8 (2.2-3.8)	
Pizza	240 (222-255)	4.1 (3.2-5.2)	516 (430-592)	2.6 (2.0-3.4)	
Pre-prepared salads and sandwich	193 (149-242)	2.3 (1.1-3.5)	408 (298-559)	3.2 (1.9-5.4)	
Ready meals	239 (186-300)	3.8 (2.3-6.3)	492 (350-618)	2.4 (1.4-4.7)	
Ready-made breakfast meals	146 (110-207)	1.6 (0.7-3.1)	353 (251-488)	1.8 (0.9-3.1)	
Soup	82 (53-326)	0.0 (0.0-0.8)	314 (185-664)	2.0 (0.8-3.7)	
Other frozen foods	259 (225-271)	1.8 (1.7-2.8)	400 (324-506)	1.1 (0.0-1.2)	
**Edible oils**	**800 (714-857)**	**13.3 (10.7-21.4)**	**0 (0-0)**	**0.0 (0.0-0.0)**	
Cooking oils	800 (800-857)	13.3 (7.1-14.3)	0 (0-0)	0.0 (0.0-0.0)	
Edible oils	714 (553-714)	32.1 (14.3-50.0)	643 (0-714)	0.0 (0.0-0.0)	
**Foods for specific dietary use**	**380 (216-417)**	**4.0 (0.5-6.7)**	**240 (84-380)**	**15.0 (5.1-26.9)**	
Breakfast beverages	97 (61-361)	0.3 (0.0-0.4)	78 (59-250)	6.3 (5.5-50.0)	
Diet drink mixes	58 (55-61)	0.3 (0.3-0.5)	65 (55-72)	5.5 (1.8-5.5)	
Protein and diet bars	400 (375-429)	5.6 (3.7-7.5)	311 (208-400)	21.7 (11.1-29.4)	
Sports gels	268 (216-294)	0.0 (0.0-0.0)	176 (133-488)	24.4 (16.2-35.3)	
Other fitness or diet products	82 (52-148)	0.4 (0.2-0.4)	79 (56-93)	5.1 (1.2-8.4)	
**Dairy**	**206 (88-321)**	**6.0 (0.7-13.3)**	**88 (50-563)**	**7.5 (0.0-19.4)**	
Cheese	357 (286-393)	16.7 (12.5-19.1)	643 (536-800)	0.0 (0.0-3.5)	
Cream	200 (133-250)	10.0 (6.7-13.3)	67 (33-100)	3.3 (3.3-6.7)	
Desserts	158 (109-359)	1.8 (1.0-6.6)	147 (118-250)	17.7 (14.1-29.6)	
Ice cream and edible ices	212 (161-246)	6.2 (2.5-7.7)	73 (51-104)	21.7 (18.8-24.3)	
Milk	59 (39-113)	0.6 (0.0-2.1)	52 (33-70)	5.0 (3.8-11.3)	
Yogurt and yogurt drinks	82 (66-94)	0.4 (0.0-1.0)	47 (37-56)	10.0 (7.1-12.7)	
**Snack foods**	**500 (438-536)**	**3.6 (1.8-7.1)**	**607 (411-893)**	**3.6 (0.0-7.1)**	
**Non-alcoholic beverages**	**40 (12-51)**	**0.0 (0.0-0.0)**	**8 (0-17)**	**8.3 (0.4-11.8)**	

Beverage mixes	357 (238-400)	0.0 (0.0-1.4)	150 (0-700)	33.3 (0.0-71.1)
Coffee and tea	351 (250-473)	6.7 (0.0-16.7)	15 (0-455)	0.4 (0.0-46.7)
Cordials	188 (49-279)	0.0 (0.0-0.0)	14 (1-34)	39.9 (11.4-61.9)
Energy drinks	24 (3-45)	0.0 (0.0-0.0)	34 (14-63)	5.6 (0.0-10.8)
Fruit and vegetable juices	46 (29-50)	0.0 (0.0-0.0)	8 (3-15)	9.6 (5.8-11.7)
Soft drinks / sodas	39 (20-48)	0.0 (0.0-0.0)	8 (2-12)	10.0 (5.0-12.1)
Sports drinks	23 (10-24)	0.0 (0.0-0.0)	45 (33-46)	5.8 (2.3-5.9)
Waters	0 (0-8)	0.0 (0.0-0.0)	0 (0-7)	0.0 (0.0-1.7)
Other beverages	29 (14-46)	0.0 (0.0-0.0)	8 (2-23)	5.8 (2.9-10.0)
**Sauces, dressings, spreads and dips**	**125 (50-264)**	**0.0 (0.0-2.9)**	**567 (342-967)**	**5.6 (1.7-17.2)**
Mayonnaise and salad dressings	267 (133-433)	3.3 (0.0-6.3)	667 (179-933)	6.7 (0.0-16.7)
Sauces	100 (48-167)	0.0 (0.0-0.0)	702 (375-1357)	6.1 (0.4-20.0)
Spreads	143 (36-333)	0.0 (0.0-6.3)	438 (286-607)	3.6 (3.2-9.4)
**Meat and meat alternatives**	**232 (143-307)**	**4.2 (0.9-8.9)**	**788 (509-1107)**	**1.1 (0.0-2.9)**
Meat alternatives	167 (123-209)	0.7 (0.0-1.4)	412 (198-538)	1.5 (0.0-2.8)
Processed meat	241 (143-313)	4.7 (1.3-8.9)	814 (554-1143)	1.0 (0.0-2.9)
**Bread and bakery products**	**386 (284-447)**	**3.6 (0.0-7.9)**	**389 (267-526)**	**20.0 (4.7-34.3)**
Bread	268 (247-295)	0.0 (0.0-1.0)	465 (388-543)	4.3 (1.8-7.1)
Cakes, muffins and pastries	372 (321-411)	5.1 (2.2-8.0)	340 (250-500)	28.1 (16.3-40.0)
Crackers and cookies	462 (421-500)	6.7 (3.1-10.7)	344 (224-500)	28.6 (10.7-36.7)
**Sugars, honey and related products**	**333 (267-375)**	**0.0 (0.0-0.0)**	**0 (0-56)**	**66.7 (45.0-82.9)**
Dessert additions	375 (278-417)	0.0 (0.0-8.6)	0 (0-83)	55.4 (33.3-70.2)
Dessert toppings	375 (325-525)	4.7 (0.0-16.2)	67 (0-158)	48.6 (34.5-67.5)
Honey	286 (286-300)	0.0 (0.0-0.0)	0 (0-0)	76.2 (76.2-76.2)
Sugar	375 (375-375)	0.0 (0.0-0.0)	0 (0-0)	100.0 (100.0-100.0)
Sweeteners	0 (0-0)	0.0 (0.0-0.0)	0 (0-0)	0.0 (0.0-99.0)
Syrup	321 (233-350)	0.0 (0.0-0.0)	25 (8-117)	56.7 (43.3-80.0)
**Confectionery**	**400 (350-500)**	**2.5 (0.0-16.7)**	**60 (12-128)**	**54.8 (45.2-66.7)**
Chocolate and sweets	400 (350-500)	3.6 (0.0-17.1)	58 (5-121)	54.8 (45.7-66.7)
Jelly	364 (81-381)	0.0 (0.0-0.0)	364 (48-429)	66.7 (0.0-87.0)

1. Deciles are calculated indvidually by nutrient among all 230,156 food and beverage products. Color for each major category and nutrient are applied based on the decile of the median.

Figure S3. Distribution of energy, saturated fat, sodium, and total sugars by major category in the US food and beverage supply.

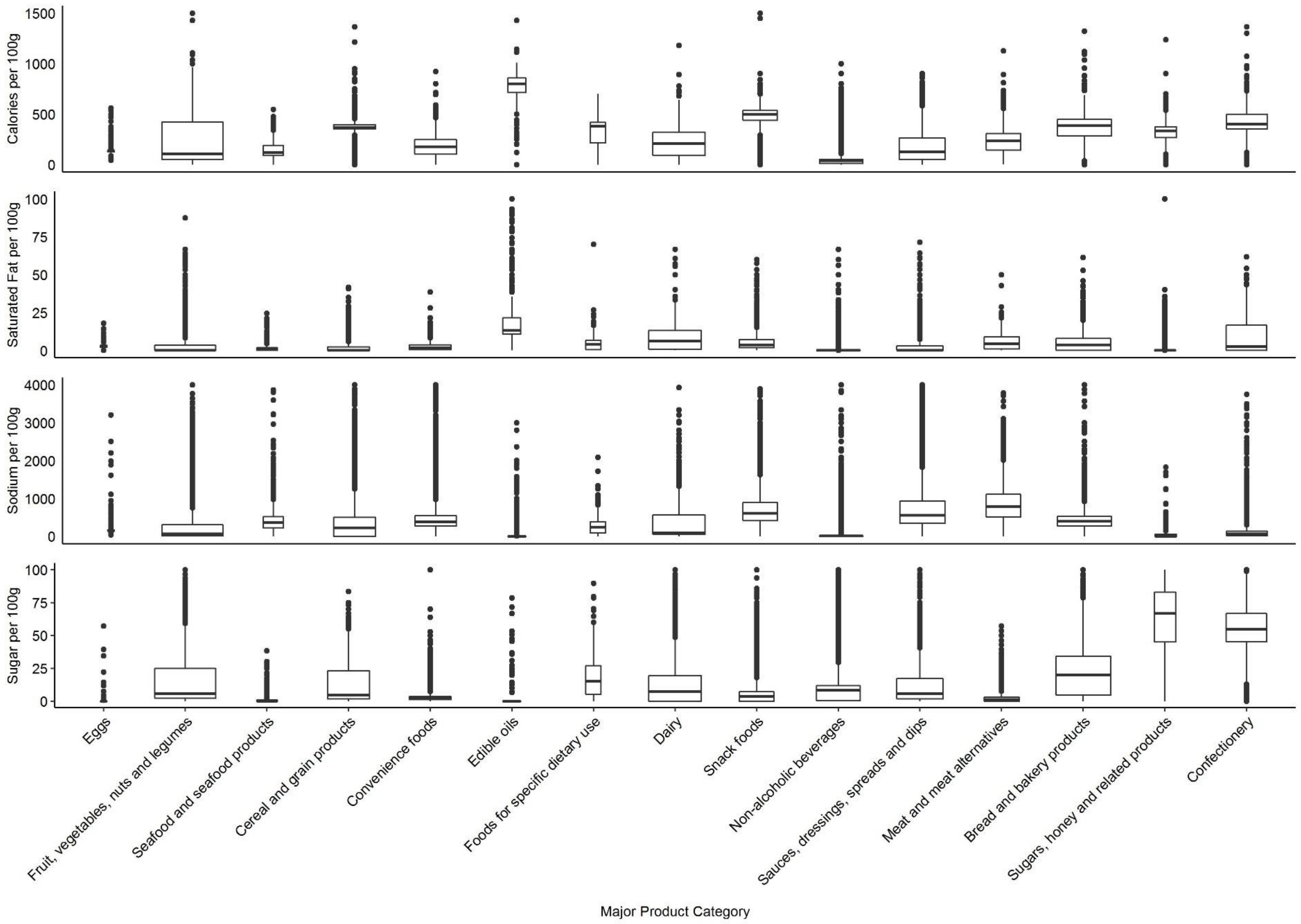

Supplement: Supplementary file 1 [file nutrients-11-01704-s001.pdf]
